# The BHU bicentric bipolar prosthesis in fracture neck femur in active elderly

**DOI:** 10.1186/1752-2897-2-7

**Published:** 2008-09-25

**Authors:** Anil K Rai, Rakesh Agarwal, Saurabh Singh, Ratnav Ratan

**Affiliations:** 1 Department of Orthopaedics, Institute of Medical Sciences, Banaras Hindu University, Varanasi-221005, India

## Abstract

**Background:**

55 BHU bicentric bipolar hemiarthroplasties were reviewed after a mean follow up of 4 years (range 1–5 years). Patients with displaced subcapital fractures were selected for operation on the basis of good mobility before the fracture. Object of the study was to see the efficacy of BHU bipolar prostheses and functional outcome.

**Results:**

There were no incidences of dislocation. Modified Harris hip scoring system scoring system was used which included sitting crosslegged and squatting in view of the sociocultural needs of the patients of Indian subcontinent. Modified Harris hip scoring system 89% had a good or excellent result and 94% had no or only occasional pain. Majority of the patients returned to their prefracture activity.

**Conclusion:**

Thus at follow up of 4 year the BHU bicentric bipolar prosthesis has been shown to be a good option for intracapsular fractures of neck femur with encouraging results.

## Background

The treatment of displaced subcapital femoral fractures in the elderly remains controversial [1,2]. Prosthetic replacement of the femoral head with the Austin Moore or Thompson hemiarthroplasties has undoubtedly played an important role in the treatment of these fractures in those who require immediate mobilisation with full weight bearing [3]. However, acetabular erosion is a significant long term complication of one piece hemiarthroplasties and is particularly common in active patients.

To abate these problems bipolar prosthesis was designed which consists of polished femoral head prosthesis with a locking internal ultra high molecular weight polyethylene (UHMWPE) bearing that mates with the head of a conventional femoral component. The rationale was that the erosion and protrusio of the acetabulum would be reduced because motion is present between the metal head and the polyethylene socket (inner bearing) thus reducing friction between the outer head and acetabulum.

**Figure 1:**
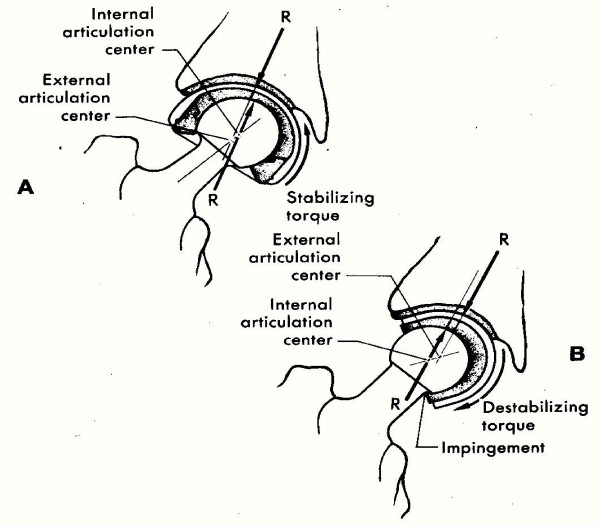
Anti varus mechanism (bicentricity).

A major advance in the bipolar cup design was making the axis of the metallic and polyethylene cups eccentric (figure 1) so that with loading of the hip, the metallic cup rotates laterally rather than medially and thus avoids fixation in varus position, as well as impingement of the head on the edge of the cup, which causes fracture of the polyethylene insert and dislocation of the implant. This concept is the essence of the BHU bipolar prosthesis. All active elderly patients presenting with a displaced sub-capital femoral fracture were treated by endoprosthetic replacement with the BHU bipolar prosthesis. This study was undertaken to audit the efficacy of this newly designed prostheses and help the patients to return to their previous normal lifestyle.

## Materials and methods

The BHU bicentric bipolar endoprosthesis was designed at the department of orthopaedics, BHU, Varanasi, India in 2002. This design evolved in an effort to palliate certain limitations of the presently used bipolar prosthesis.

**Figure 2:**
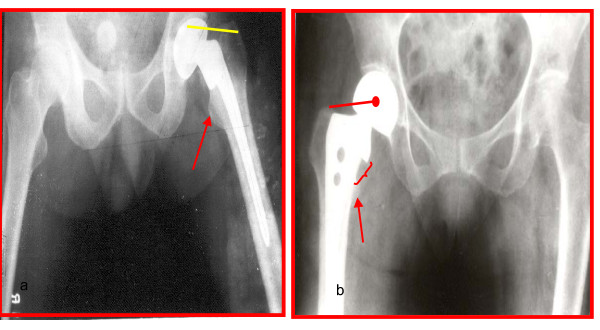
**Unacceptably low angle and disadvantage of sacrificing the important calcar has been dealt with by making the angle as anatomical as possible**. a) Conventional bipolar b) BHU bipolar.

**Figure 3:**
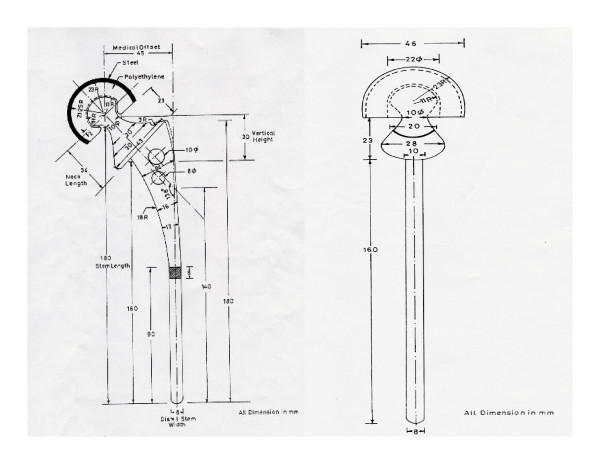
BHU bicentric diagramatic presentation.

**Figure 4:**
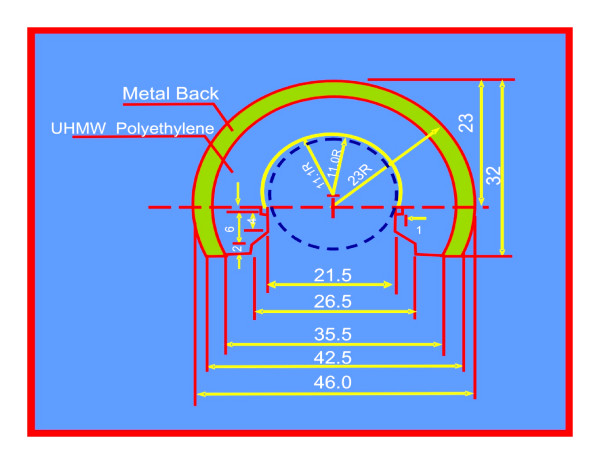
UHMWPE cup with bevelled margin (staggering) to avoid neck cup impingement.

**Figure 5:**
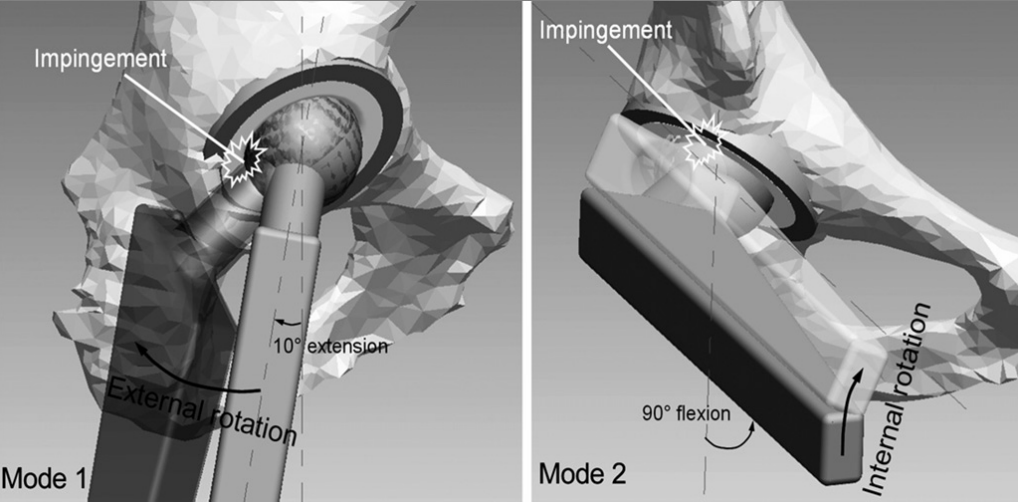
Neck cup impingement.

**Figure 6:**
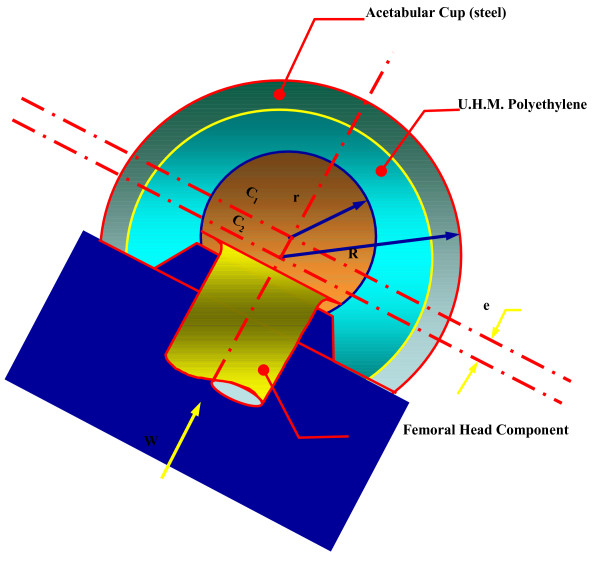
Mt = (p.W/2).μ.r for given load W, the frictional torque is proportional to the product of coefficient of friction and radius of hemispherical surface.

**Figure 7:**
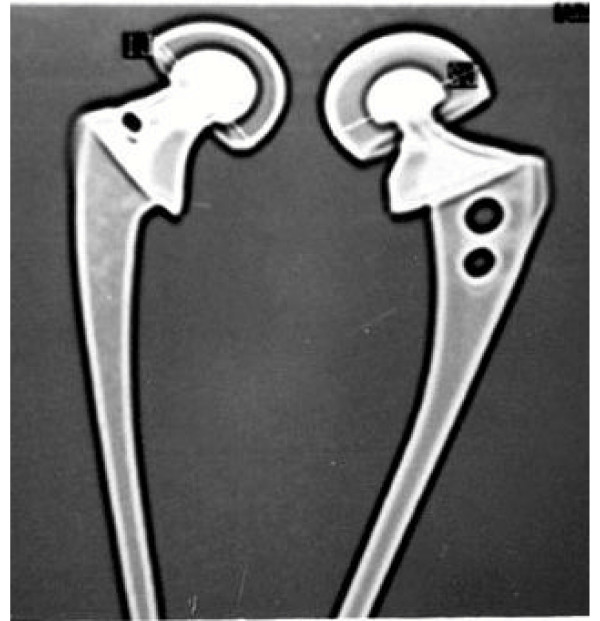
BHU bipolar prosthesis (right side) showing increased thickness of UHMWPE due to smaller diameter of the inner head.

**Figure 8:**
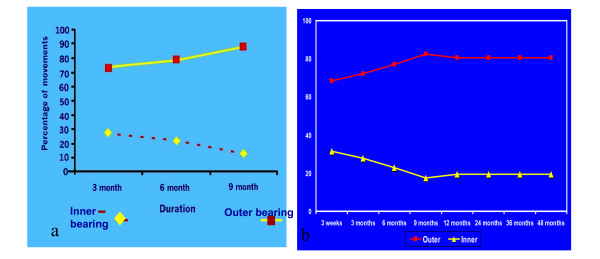
**Graphic diagram for inner and outer movement with the duration of time**. a) In a study done on Conventional bipolar, it was found that the movements occurring at the inner interface gradually decrease with time. b) BHU hip device showed decrease in inner movement initially but in long term there was continuous movement in inner bearing *p value *not signifiant.

**Figure 9:**
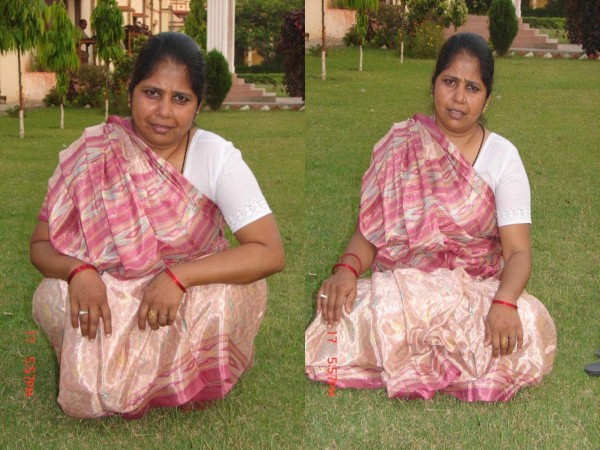
Patient in squatting and sitting crosslegged position.

1. The seating angle between the stem and the collar has been modified so that after seating of the prosthesis there is preservation of the calcar by quarter to one cm. In conventional bipolar this angle is so low that the calcar has to be sacrificed for seating and hence on load bearing it sinks, if cement is not used (figure 2 &3).

2. The UHMWPE lining has a bevelled margin (figure 4). Thus at the extremes of movement, the neck impinges on the metal cup and not on the UHMWPE lining therefore reducing wear and debris formation. Neck cup impingement is a very important factor for the failure of bipolar prosthesis. To tackle this problem the cup has been modified in staggering fashion and the neck of the prosthesis has been made trapezoidal like Charnley type neck (figure 3). This change in design has provided overall gross increase in the movement at the inner articulation and avoids the neck cup impingement, a very important cause for the failure of the bipolar prosthesis (figure 5) [4].

3. The movement of the inner bearing under the plastic cup is dependent on the frictional torque difference between the outer and inner articulation. The outer articulation is the acetabulum and bipolar cup and inner articulation between UHMWPE insert and the stainless steel head. As per mathematical calculations, the frictional torque is dependent on radius of the circle [(Mt = (π.W/2).μ.r] for given load W, the frictional torque is proportional to the product of coefficient of friction and radius of hemispherical surface (figure 6). Hence the smaller diameter head will move better into the cup and thus provides a better positive eccentricity. For this reason in the BHU bicentric bipolar has the Charnley head design.

4. The smaller diameter head provides a larger thickness of UHMWPE for the better survivorship of the prosthesis. UHMWPE wear in a bipolar is 0.7 mm./year [5]. For this very reason, a 22 mm. Charnley head diameter has been in the BHU bicentric bipolar prosthesis (figure 7).

5. It has been reported that with duration of time the outer movement between the acetabulum and the cup increases and the inner movement between the UHMWPE and the stainless steel head decreases and the bipolar becomes unipolar with the change in the design in the neck and the cup (staggered margin) there is preservation of 70% of the total movement at the inner bearing after four years follow up (figure 8).

6. For social and cultural reasons sitting and squatting is the integral part of daily life style of the patient of Indian subcontinent. The bicentric bipolar prosthesis provides the same without limiting patient range of movement at hip (figure 9).

7. It is available in 37 to 55 head sizes in 1 mm increments, which prevents use of inappropriate head sizes and subsequently early failure of the prosthesis. This avoids the Hertzian contact stress [6].

8. The design includes a cemented and an uncemented variety of stem.

9. The benefit of positive eccentricity has been provided by reducing the head diameter of the inner ball to 22 mm. This also provides a greater range of movements between the metallic head and the UHMWPE, thus providing greater percentage of movement at the inner bearing for a longer time adding longevity to the system.

This study evaluated, (i) The range of motion between the inner and outer bearing of BHU bipolar prostheses and its change with time (ii) The clinical outcome after hemiarthroplasty with this bipolar system and its change on subsequent follow-ups. Between april 2003 and jan 2008, 55 BHU bipolar hemiarthroplasties were performed in 55 patients; 90.9% were fresh fractures. There were 35 females and 20 males with an average age of 60 years (range 45–90 years). Patients were selected for the operation on the basis of their mobility before injury and their level of independence. Prior to admission 45 patients were walking independently and 10 (18.18%) were using one stick. The results were evaluated using Merle d Aubigne postal system and Modified Harris Hip scoring system (Table 1)[7]. Finally the results of the movement at the inner and outer bearings of bipolar prosthesis, and its functional results were obtained. Under antibiotic cover, the majority of operations were performed via the lateral Hardiange approach; all the surgeons were of consultant or senior registrar grade.

**Table 1:** The Clinical Rating System

**(a) *HIP Assessment***		**(b) *Patient Function***	
**Pain**	**Points**	**Walking Aids**	**Point**

None	25	None	20
Occasion	10	01 Stick	15
Moderate	05	02 Stick/Frame	10
Severe	0	Immobile	0
			
**Range of Motion**		**Sitting Cross Leggged**	**10**
Normal	15	Squatting	10
Moderate Reduction	10	Cannot Sit Cross Leg	0
Severe Reduction	0	Cannot squat	0
		**Patient Satisfaction**	
**Positive Trendelenberg**	**-15**	Very Good	20
		Good	10
		Fair	05
		Poor	0
			
**Maximum Total**	**40**	**Maximum Total**	**60**

A Potential score according to pre-op mobility, for example if patient was using 1 stick prior to fracture and also at the time of review, they were awarded 15 points. Grades awarded according to total marks. Excellent = 100-81 points; good = 80-61; fair 60-41 and less than 40 = failure.

## Clinical Evaluation

Modified Harris hip rating system was devised to allow analysis of the overall function of the patient (Table 1) with special emphasis on sitting crosslegged and squatting postoperatively to meet out the sociocultural needs of the patients of Indian subcontinent Scoring was heavily weighted towards a painless hip which allowed normal motion and activity. A positive Trendelenberg sign was marked negatively and attracted a maximum grade of fair. The patient's subjective analysis of the operation was also recorded. All revisions and dislocations were classed as failures, and telephone reviews were allowed a maximum grade of good.

## Results

Overall the operation was well tolerated with 45 patients discharged home by 12 days and 10 by 3 weeks. Post-operative complications included deep vein thrombosis 3, pulmonary embolus 1, and chest infection 4. There were 3 cases of superficial wound infection, all successfully treated, and there were no deep infections. No dislocations had occurred till last follow up. There were 4 mortalities in early follow up (within 1 yr) due to chest infection and pulmonary embolus.

Of the 51 patients surviving, 1 was untraceable, 47 were available for clinical and radiographic review and 3 answered a telephone questionnaire till last follow up 49 (89%) patients had a satisfactory result with an excellent or good clinical grade (Table 2). 52 patients had no or slight pain, not requiring analgesia. 47 (85.45%) patients could sit crosslegged and squat (figure 9) and could use the squatting toilet. They were almost pain free and leading normal life at the end of 4 yr follow up.

**Table 2:** Clinical Grade

Excellent	40(72.72%)
Good	9(16.36%)
Fair	4(7.2%)
Poor	2 (3.64%)
Total No.	55

The operation was rated as very good or good by 49 patients. The 4 patients with a fair grade had a positive Trendelenberg sign but were otherwise free of pain and expressed satisfaction with the result of their operation. Of two failures, both had sinkage of prosthesis due to poor bone stock. They all developed severe pain in the early postoperative phase and were revised to total hip arthroplasty.

Total movement was on an average 23.8°. This improved slightly at 6 months and was almost same as on the last postoperative follow-up by the 48 month (mean 36.86°). Movement at the outer bearing gradually increased from a mean of 16.27 (68.36%) to a mean of 19.21° (80.51%) from the 1 to last follow-up. The movements at the inner bearing gradually decreased from a mean of 7.53° (31.64%) to 4.65° (19.49%). Analyses of these movements were carried out and student 't' test applied. It was found that there was no significant difference in the total abduction movement from 1st to last follow-up. There was no significant difference in the movement of the outer bearing from the 3 week till 36 months post operatively. Similarly, there is no significant difference between movements of the inner bearing with the passage of time (figure 8).

Therefore, although there was a slight loss of movement at the inner bearing over the months on subsequent follow up as compared to the 1st follow-up, this difference was not statistically significant.

## Discussion

Experience has shown that the acetabulum can tolerate only limited activity following a one-piece hemiarthroplasty if degenerative changes are to be avoided. In one of the longest follow up reports for the cemented Thompson's prosthesis, Kuokkouen, Souminer and Korkala reviewed 78 patients 10 years after their operation [8]. They found that 24 had already been converted to total hip replacement; in 23 of the remaining 54 the prosthesis was radiographically loose and there was severe protrusio in 25 cases. The authors concluded that the Thompson hemiarthroplasty should be restricted to patients with a short life expectancy. Although radiographic acetabular measurements are inaccurate, we did not find any evidence of acetabular wear in any patient, and no prostheses appeared radiographically loose or had been revised for aseptic loosening. The patients in the present series were very active with over 90% walking with no or one walking aid and over 90% living independently before admission.

Our findings are consistent with previous studies of cemented bipolar prosthesis in which no cases of protrusio have been reported, including one report with an average follow up of 7 years and 5 months [9].

These figures compare very favourably with reports of one-piece hemiarthroplasties with similar follow up. Phillips using a cemented Thompson prosthesis, reported acetabular erosion in 34 out of 38 hips in active patients at an average postoperative period of 7.7 years [10].

In a group of patients treated with the Austin-Moore prosthesis, Kofoed and Kofod reported a 55% incidence of painful acetabular erosion requiring conversion to a total hip replacement after only 2 years [11].

The low mortality reported in the present series of 7.27% at 12 months is considerably less compared with 23% reported by D' Arcy and Devasis a reflection of the policy of patient selection and reasonable life expectancy of these patients[12]. We believe that pre-fracture mobility and an independent existence are more predictive of life expectancy than the patient age [4].

Using modified Harris hip scoring system 89% of patients had a good or excellent result with an average follow up of 4 years. Pain was absent or slight in 94% of patients at review. This figure is similar to that reported by Bochner et al and Wetherell et al [13,14]. After operation three patients had severe pain, all presenting in the early postoperative phase. Two were subsequently converted to total hip replacement, which is a relatively straightforward procedure; adequate acetabular exposure can be obtained at operation by removing the bipolar component and the femoral head.

The aim of surgery was to restore normal hip anatomy. Traditional one-piece prostheses lack the range in head size required to consistently achieve an anatomical replacement. In a radiographic analysis of 599 one-piece hemiarthroplasties, Kwok and Cruess found an inappropriate neck length in 55% and an incorrect head size in 25% of cases [15]. They postulated that this may lead to uneven and increased stress at the hip joint with resultant accelerated acetabular wear.

Following the introduction of the bipolar design, reports of interprosthetic separation began to appear, which tempered the early enthusiasm for this type of implant [16,17]. The early bipolar models tended to assume an extreme varus position on loading resulting in rapid acetabular wear and prosthetic separation. This problem has been largely solved by incorporating a small polar offset between the outer head centre and inner bearing centre which generates a more stable valgus-producing moment on loading [18]. This property is known as "positive eccentricity" or "anti-varus dynamics". There were no incidences of prosthetic dislocation in the total 55 patients.

Functional capacity in the present study was assessed by the modified Harris hip scoring system in which were incorporated elements of ability to sit cross legged, ability to squat and patient satisfaction. As mentioned earlier certain components not relevant to our Indian patients were ignored. Almost all the patients showed remarkable improvement in the modified Harris hip score. This compares favourably to numerous other studies.

## Conclusion

Thus at follow up of 4 years the BHU bicentric bipolar endoprosthesis has been shown to be a good option for intracapsular fractures of neck femur with encouraging results.

Sitting cross-legged and squatting are contraindicated after total hip replacement and unipolar hemiarthroplasty. Indian subcontinent patients due to their lifestyle and customs are greatly handicapped if they are unable to do these movements. They have to modify their lifestyle to be able to carry out their basic daily activities. With the BHU bicentric bipolar, patients were very much satisfied to go back to their previous lifestyle without too many difficulties and caters to their daily.

## Abbreviations

BHU: Banaras Hindu University; UHMWPE: Ultra high molecular weight polyethylene.

## Competing interests

No fees, funding or grants has been received for development, designing and clinical trial for this bicentric bipolar prosthesis.

## Authors' contributions

AR conceptualized the design and the clinical trial of the prostheses and holds national patent of the design under the name of BHU hip. RA and RR did the clinical study and compiled all the data to analyse the outcome. RA helped in preparing the manuscript and reviewing. SS thoroughly reviewed the manuscript.

## Consent statement

Prior written consent was taken from the patient for  publication of  picture in the article.
